# Assessment of the canal anatomy of the premolar teeth in a selected Turkish population: a cone-beam computed tomography study

**DOI:** 10.1186/s12903-023-03107-7

**Published:** 2023-06-19

**Authors:** Erhan Erkan, Keziban Olcay, Tan Fırat Eyüboğlu, Elif Şener, Mustafa Gündoğar

**Affiliations:** 1 Department of Endodontics, School of Dentistry, Medipol Mega Dental Hospital, Istanbul Medipol University, TEM Avrupa Otoyolu Goztepe No: 1, Bagcilar, Istanbul, 34214 Turkey; 2 Department of Endodontics School of Dentistry, Istanbul University-Cerrahpasa, Kocamustafapasa Cad. No: 34/E, Cerrahpasa, Fatih, Istanbul, 34098 Turkey; 3 Department of Endodontics, School of Dentistry, Istanbul Medipol University, Ataturk Blvd., No: 27, Unkapanı, Fatih, Istanbul, 34083 Turkey; 4 Department of Oral and Maxillofacial Radiology, School of Dentistry, Ege University, Erzene Mah. Ankara Cad. No: 172/109, Bornova, Izmir, 35040 Turkey

**Keywords:** Maxillary premolars, Mandibular premolars, Cone-beam CT, Root canal morphology

## Abstract

**Background:**

Teeth may have additional roots and a different number of root canals. Overlooked root canals may cause endodontic failure. The aim of this study was to investigate the prevalence of root canals and the number of roots of premolars in a selected Turkish population.

**Materials and methods:**

A total of 2,570 teeth from 1,438 patients were evaluated. The cone-beam computed tomography scans of 1,055 maxillary and 1,515 mandibular premolars were examined.

**Results:**

Type IV root canal morphology was observed most frequently in maxillary first premolars (77%), and the rates of single and double channel formations were very similar (51% and 49%, respectively). Of the second maxillary premolars, 57.4% had Type I morphology, and 89.9% of the teeth were single-rooted, while 68.6% had a single root canal. The most common formation was Type I (85%) among mandibular first premolars, and a single root was observed in 95.6% of these teeth. In addition, 87% of the mandibular first premolars had a single root canal. The second mandibular premolars mostly had Type I (95.4%) formation, and 99.3% of the teeth were single-rooted, while 96.9% had a single root canal.

**Conclusion:**

According to our findings, 51% of maxillary first premolars had a single root, 79.4% had two root canals, and 77% had Type IV (77%) formation. Maxillary second premolars mostly had Type I formation. In addition, a single root and single root canal formation were most common. Mandibular first premolars generally had a single root and single root canal formation, but 13% had two root canals, and 6.4% had Type V formation. More than 95% of mandibular second premolars had Type I formation.

## Introduction

It can be challenging for dentists to perform adequate shaping and disinfection of root canals due to the complex structure and diversity of root canal anatomy. Good knowledge of root and root canal morphology is important and essential for successful long-term results and a good prognosis [1]. Vertucci described a classification system consisting of eight types to demonstrate the morphology of pulp and canal formation [2]. Since then, other researchers have defined further subgroups for this classification [3, 4].

In recent years, various techniques have been described to explore root canal complexes, such as staining and clearing, radiography techniques, and micro-computed tomography (µCT) imaging [5–9]. Traditional and periapical radiographs produce two-dimensional (2D) images. However, due to distortions caused by superimposition on these images, 2D imaging systems do not reflect the complete morphology of root canals, especially in the premolar and molar regions [10]. µCT is a newly used and non-destructive method in the dental field, providing detailed quantitative and qualitative measurements at high resolution for anatomic studies. µCT shows the complex anatomy and allows for an accurate 2D or three-dimensional (3D) assessment of the root canal system. Except for conventional 2D imaging systems that evaluate root canal anatomy, all these methods have diagnostic accuracy only in extracted teeth and cannot be used in the clinical setting [11]. Therefore, cone-beam computed tomography (CBCT) presents as a promising modality due to its high imaging quality and non-invasive methodology [12], which has improved the detection of additional roots and canals through 0.125-2 mm sections taken in the axial, coronal, and sagittal planes. Over the last decade, CBCT has facilitated diagnoses in endodontics and provided clinicians with 3D information to better understand the thorough morphology of root canals by eliminating superimposition, which is an integral part of conventional radiographic imaging [12].

According to the findings of previous studies, premolar teeth have extremely variable root and canal morphologies according to race and geographic origin [13, 14]. There are many studies in the literature evaluating the root canal complex of premolar teeth using CBCT images. However, most of these studies have included only premolars in the maxilla or mandibula, and there is only limited research concerning the root canal morphology of premolar teeth in both structures [15–22]. Therefore, the current study was planned to investigate the root canal complex of premolar teeth in a selected local Turkish population according to gender and age using CBCT.

## Materials and methods

This study was approved by the ethical board of the university (approval number: E-10840098-772.02-193). A total of 2,570 mandibular and maxillary premolar teeth, for which CBCT images were taken for surgical dental operation planning between 2000 and 2017 at the Faculty of Dentistry, were evaluated. The digital radiographic images of the patients were obtained from the hospital’s database. The patients’ personal information was also recorded. Premolar teeth of good periapical health that had complete root formation and had not received any dental treatment were included in the study. Images with digital defects were excluded.

The CBCT images of the patients were taken using an i-CAT17–19 imaging system (Imaging Sciences Int., Inc.) with a standardized scanning protocol and a voxel size of 0.25 mm, according to the manufacturer’s recommendations. All volumes were acquired at 120 kVp and 20.27 mAs using a 16 cm × 11 cm field of view.

Morphologies were examined by two endodontists and one radiologist with at least 10 years of experience. In order to calibrate the observers, 10 of the obtained data points were randomly selected and examined by two endodontists twice at 10-day intervals. In cases where a different decision existed, a consensus was reached further by consulting an oral diagnosis and radiology specialist. Cohen’s Kappa coefficient for the interobserver agreement was determined as 0.75.

**Fig. 1:**
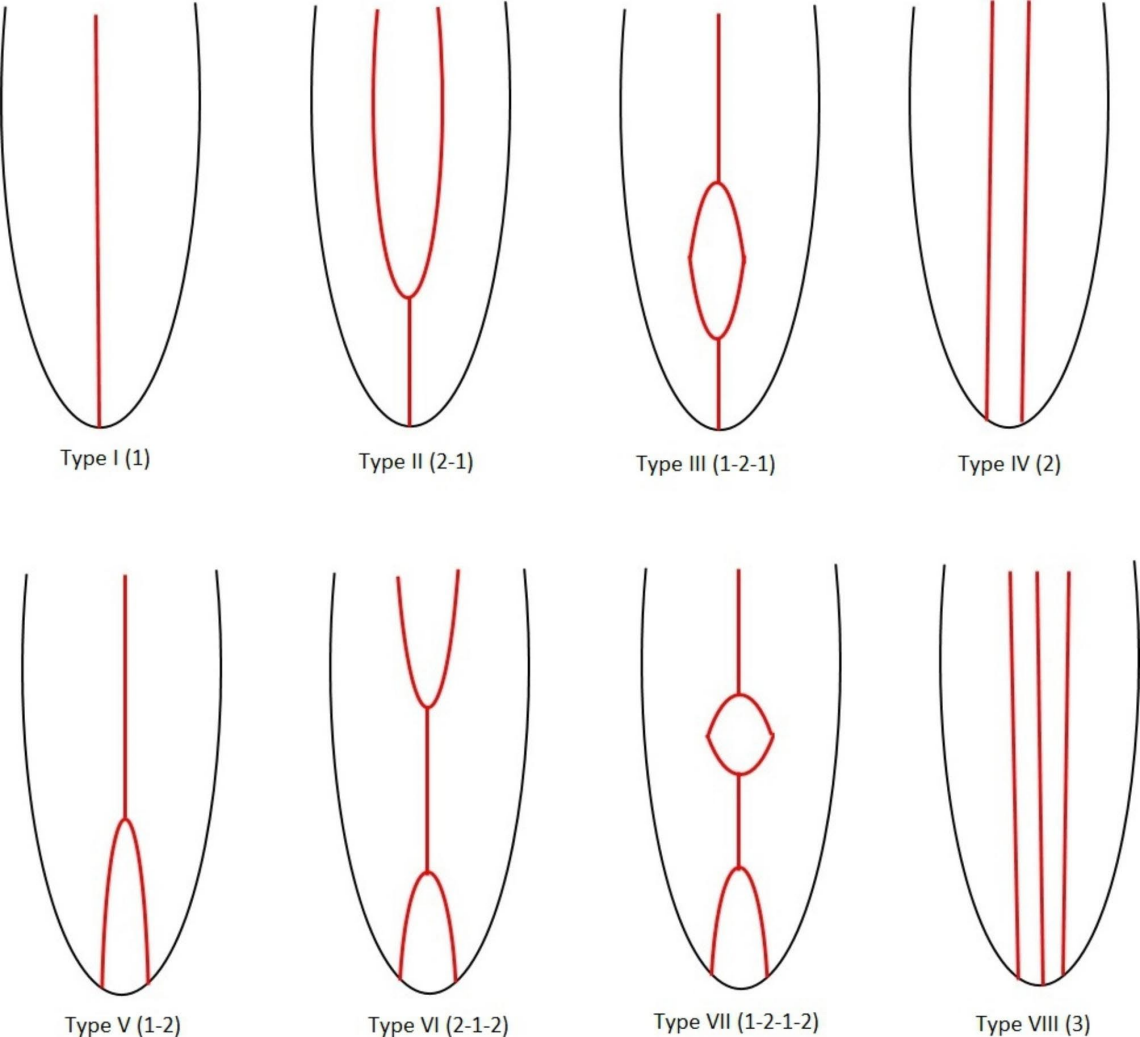
Vertucci Classification of Root Canal Morphology

A series of images were viewed from the cementoenamel junction to the root apex, and the root canal complex was classified according to the Vertucci classification shown in Fig. 1.

### Statistical analysis

IBM SPSS Statistics v. 22 software (IBM SPSS, Turkey) was used to analyze the data obtained from the study. In the comparison of qualitative data and analysis of differences according to gender, the chi-square test was used. The differences between the parameters were evaluated to be statistically significant when the *p* value was < 0.05.

## Results

The examination was carried out on 2,570 teeth of 1,438 patients, of whom 604 (42%) were male and 834 (58%) female, with an age range of 13 to 81 years. The ratio of women was significantly higher than that of men (p < 0.05). The mean age was 40.02 ± 13.63 years. The ages of the men varied between 13 and 81 years, with a mean value of 41.17 ± 13.91 years. The ages of the women varied between 16 and 74, with an average value of 39.18 ± 13.37 years. The number of maxillary teeth was 1,055 (41.1%), and the number of mandibular teeth was 1,515 (58.9%). Of the teeth examined, 1,282 (49.9%) were on the right side, and 1,288 (50.1%) were on the left. In addition, 521 (49.4%) teeth were on the right side of the maxilla, and 534 (50.6%) were on the left side of the maxilla, while 761 (50.2%) teeth were on the right side of the mandibula, and 754 (49.8%) were on the left side of the mandibula (Table 1).

**Table 1:** Canal Configurations of Premolar Teeth by Gender

Gender		Men (n = 1,065)	Women (n = 1,505)	Total (n = 2,570)	*p*
Root Number	One root	886 (83.2%)	1,327 (88.2%)	2,213 (86.1%)	0.000*
	Two roots	179 (16.8%)	178 (11.8%)	357 (13.9%)	
Root Canal Number	One	760 (71.4%)	1,092 (72.6%)	1,852 (72.1%)	0.505
	Two	305 (28.6%)	413 (27.4%)	718 (27.9%)	
Vertucci Classification	Type I	689 (64.7%)	1023 (68%)	1,712 (66.6%)	0.022*
	Type II	48 (4.5%)	43 (2.9%)	91 (3.5%)	
	Type III	25 (2.3%)	28 (1.9%)	53 (2.1%)	
	Type IV	255 (23.9%)	360 (23.9%)	615 (23.9%)	
	Type V	34 (3.2%)	42 (2.8%)	76 (3%)	
	Type VI	2 (0.2%)	5 (0.3%)	7 (0.3%)	
	Type VIII	12 (1.1%)	4 (0.3%)	16 (0.6%)	
					*p < 0.05

While 86.1% of all teeth were single-rooted, 13.9% were double-rooted. There was a statistically significant difference between the genders in terms of the number of roots (p < 0.05). The incidence of double roots in males (16.8%) was significantly higher than in females (11.8%). A single root canal was present in 72.1% of all teeth, and two root canals in 27.9%. There was no statistically significant difference between the genders in terms of the number of root canals (p > 0.05).

**Fig. 2:**
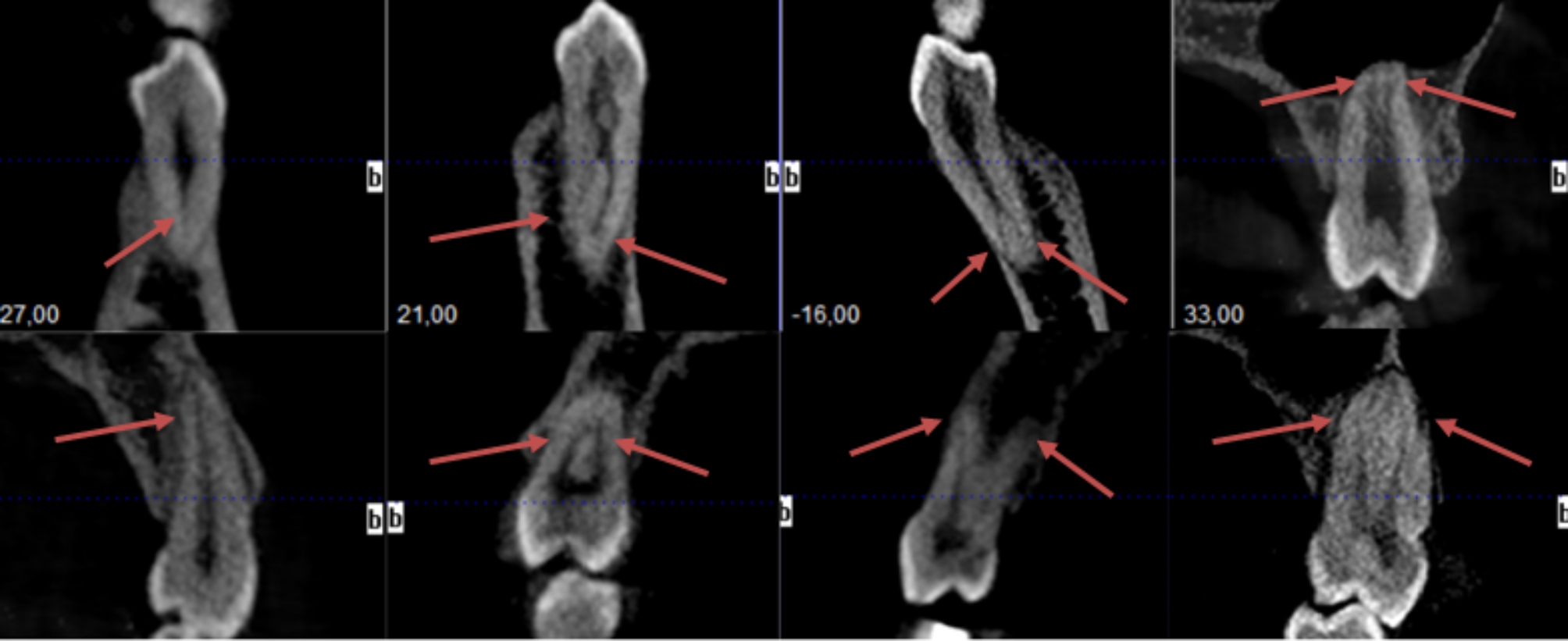
Root Canal Configurations of Premolars

Considering the distribution of Vertucci classes, of all teeth, 66.6% were Type I, 23.9% were Type IV, 3.5% were Type II, 3% were Type V, 2.1% were Type III, 0.6% were Type VIII, and 0.3% were Type VI (Fig. 2). There was a significant difference between the genders in terms of the Vertucci classification (p < 0.05). The incidence of Type I was significantly higher in women (68%) than in men (64.7%).

When the upper premolars were evaluated according to the Vertucci classification, the incidence of Type I was 10.2% in the first premolars and 57.4% in the second premolars. The incidence of type IV was 77% in the first premolars, while this rate was 28.9% for the second premolars. Type V canal configuration was observed in 0.4% of the first premolars and 1.9% of the second premolars. Type VIII formation was seen at a rate of 1.5% among the first premolars and 0.2% among the second premolars. When the mandibular premolars were classified, the incidence of Type IV was 5.7% for the mandibular first premolars and only 0.7% for the mandibular second premolars. The incidence of type V was 6.4% in the mandibular first premolars and 1.7% in the mandibular second premolars.

The most common canal formation in the maxillary first premolar teeth was Type IV (77%), and single root formation was more common (51%). Of the maxillary first premolars, 79.4% had two root canals. In the maxillary second premolar teeth, the most common canal formation was Type I (57.4%), and 89.9% of these teeth were single-rooted, while 68.6% had a single root canal.

Among the mandibular first premolar teeth, the most common canal formation was Type I (85%), 95.6% of the teeth were single-rooted, and 87% had a single root canal. When the mandibular second premolar teeth were evaluated, the most common canal formation was Type I (95.4%), and 99.3% of these teeth were single-rooted, while 96.9% had a single root canal (Table 2).

**Table 2:** Canal Configurations of Premolars according to the Vertucci classification

		Number of Roots (%)		Number of Root Canals (%)		Vertucci Classification (%)						
Tooth	n	1	2	1	2	Type I	Type II	Type III	Type IV	Type V	Type VI	Type VIII
14	268	48.9	51.1	21.6	78.4	10.8	6	5.2	75,0.4	0.4	0	2.2
24	271	53.1	46.9	19.6	80.4	9.6	7.4	2.6	78.6	0.4	0.7	0.7
Total	539	51.0	49.0	20.6	79.4	10.2	6.7	3.9	77.0	0.4	0.4	1.5
15	253	88.9	11.1	67.6	32.4	56.9	3.2	7.5	29.6	2.4	0	0.4
25	263	90.9	9.1	69.6	30.4	57.8	8.4	3.4	28.1	1.5	0.8	0
Total	516	89.9	10.1	68.6	31.4	57.4	5.8	5.4	28.9	1.9	0.4	0.2
34	405	95.6	4.4	87.2	12.8	85.7	2	0	5.4	5.9	0.5	0.5
44	409	95.6	4.4	86.8	13.2	84.4	2.2	0.2	5.9	6.8	0	0.5
Total	814	95.6	4.4	87.0	13.0	85.0	2.1	0.1	5.7	6.4	0.2	0.5
35	349	99.7	0.3	97.4	2.6	96.3	0.9	0.3	0.3	2	0	0.3
45	352	98.9	1.1	96.3	3.7	94.6	1.4	0.6	1.1	1.4	0.3	0.6
Total	701	99.3	0.7	96.9	3.1	95.4	1.1	0.4	0.7	1.7	0.1	0.4

## Discussion

The current study evaluated the premolar teeth using CBCT in a local Turkish population. A total of 2,570 teeth belonging to 604 male (42%) and 834 female (58%) patients aged 13–81 years were included in the study, and 1,055 maxillary and 1,515 mandibular premolars were examined.

In this study, it was found that the root canal formation in the premolars was generally Vertucci Type IV (77%), followed by Type I (10.2%). The rate of Type IV formation in the maxillary premolars was higher than reported in previous studies. Vertucci reported 62% Type IV formation in the maxillary premolars, while Peiris reported this rate to range from 45.7 to 64% [2, 23]. On the other hand, our results were similar to those determined by Caliskan et al. (78.4%), and Awawdeh et al. (79.7%) [2, 14]. These similarities and discrepancies may be due to the differences between the studies in terms of the geographical area and sample.

In terms of the root canal number of maxillary first premolars, we found that double-rooted canal formation was most common (79.4%) and was seen at a higher rate than reported by Pineda and Kuttler (73.3%) [5] and lower than determined by Vertucci (87%) [2], Caliskan et al. (96.1%) [24], Kartal et al. (89.6%) [25], Sert and Bayirli (86%) [26], Awawdeh et al. (94.5%) [14], and Ok et al. (89.4%) [1]. In our study, approximately half of the maxillary first premolars (51%) had a single root. In the literature, this rate was reported to be lower by some researchers, e.g., 39.5% by Vertucci [2], 37.3% by Kartal et al. [25], and 17.9% by Atieh [7], while it was found to be higher by Peiris (76.6%) [23] and Tian et al. (66%) [12].

In our evaluation, the maxillary second premolars mostly had a single root (89.9%) and a single root canal (68.6%). Similarly, Bürklein et al. [22] reported that the maxillary second premolars in a German population mostly had a single root (82.6%), but the authors reported a higher incidence of double-rooted canals (56.3%) compared to our findings. In our study, the maxillary second premolars mostly presented with Vertucci Type I formation (57.4%), followed by Type IV (28.9%). Bürklein et al. [22] reported the most common morphological root canal types to be IV (25%) and V (28.7%) in their German sample.

In the current study, the mandibular first premolars mostly had a single root and single canal formation (85%) (Type I). This rate is higher than reported by Vertucci (70%) [2], Caliskan (64%) [24], Sert and Bayirli (60.5%) [26], Rahimi (69.4%) [27], and Awawdeh and Al-Qudah (58.2%) [28], and similar to the findings of Liao et al. (83.5%) [29] and Yu et al. (86.8%) [30].

We observed that 95.4% of the mandibular second premolar teeth had Type I formation. The second most common morphological type was Type V (1.7%). These findings are in agreement with those reported by Cleghorn et al. (99.6%) [31]. Root and canal morphologies vary across age and gender groups due to ethnic and genetic factors. The current study showed different data compared to previous studies conducted with Turkish populations [1, 24–26, 32]. Therefore, there is a need for comprehensive studies to make a more accurate interpretation of these results.

Different methodologies are used to investigate root and root canal anatomy, and they are basically divided into invasive and non-invasive techniques. µCT imaging is very effective in describing root canal anatomy but can only be performed on extracted teeth [10, 11]. CBCT imaging is another effective technique, and it has the advantages of data being accessible from any health institution and the procedure not requiring tooth extraction [11]. Considering similar studies, the use of CBCT imaging is safe and effective for performing similar evaluations [8–11].

## Conclusion

According to the evidence provided by our study, the incidence of two root canals was significantly higher in men, while the incidence of Type I canal formation was significantly higher in women. When we evaluated the root canal morphology of the four different types of premolars (maxillary first and second premolars and mandibular first and second premolars), we determined that the most common canal formation in both jaws was Type I, except in the maxillary first premolars (Type IV). We consider that the results we obtained from a total of 2,570 teeth of 1,438 patients provide a more comprehensive evaluation of the root canal complex of both the maxillary and mandibular premolar teeth than previous studies that only evaluated one jaw (maxilla or mandible) and will assist clinicians in their decisions related to optimal diagnosis and treatment planning.

## Data Availability

The files in which the data obtained from the patient images are recorded in the study are kept by the corresponding author and can be shared with the approval of the university’s ethics committee.
